# PubMed Central: offering a trove of historical medical journals

**DOI:** 10.5195/jmla.2025.2235

**Published:** 2025-10-23

**Authors:** Jeffrey S. Reznick, Kathryn Funk, Laura Randall, Katy Rose Taylor, Katherine Chan, Ellen Adams

**Affiliations:** 1 jeffrey.reznick@nih.gov, Senior Historian, National Library of Medicine, National Institutes of Health, Bethesda, MD; 2 kathryn.funk@nih.gov, Supervisory Technical Information Specialist, National Center for Biotechnology Information, National Library of Medicine, National Institutes of Health, Bethesda, MD; 3 laura.randall@nih.gov, Technical Information Specialist, National Center for Biotechnology Information, National Library of Medicine, National Institutes of Health, Bethesda, MD; 4 katy.taylor@nih.gov, Technical Information Specialist, National Center for Biotechnology Information, National Library of Medicine, National Institutes of Health, Bethesda, MD; 5 katie.chan@nih.gov, Program Analyst (Evaluation and Data), National Library of Medicine, National Institutes of Health, Bethesda, MD; 6 ellenpadams@gmail.com, BLH Technologies, Inc., Rockville, MD

**Keywords:** Biomedical journals, Historical Medical Archive, Digitization, Articles, PubMed Central

## Abstract

This article briefly documents the history and significance of PubMed Central (PMC) Journal Backfiles Digitization, 2004-2024 to raise awareness of this open access project among researchers who will find much to discover to advance understanding about the human condition across time and place. The success of PMC Journal Backfiles Digitization—including the interdisciplinary teamwork and partnerships underpinning it—provides a blueprint for future efforts to make the globally appreciated collections of the National Library of Medicine (NLM) accessible to all. By continuing to prioritize open access, teamwork, and partnerships, NLM and likeminded institutions can ensure that knowledge and data inform the advancement of medicine and public health.

In 2004, a collaboration began—eventually spanning two decades—that would measurably transform access to historical medical literature: PubMed Central (PMC) Journal Backfiles Digitization [1, 2] [Figures 1 and 2]. This ambitious project involved digitizing and making available via open access the back issues of a vast collection of medical journals rich with qualitative and quantitative data to be discovered and studied to advance understanding of the human condition across time and place.

**Figure 1:**
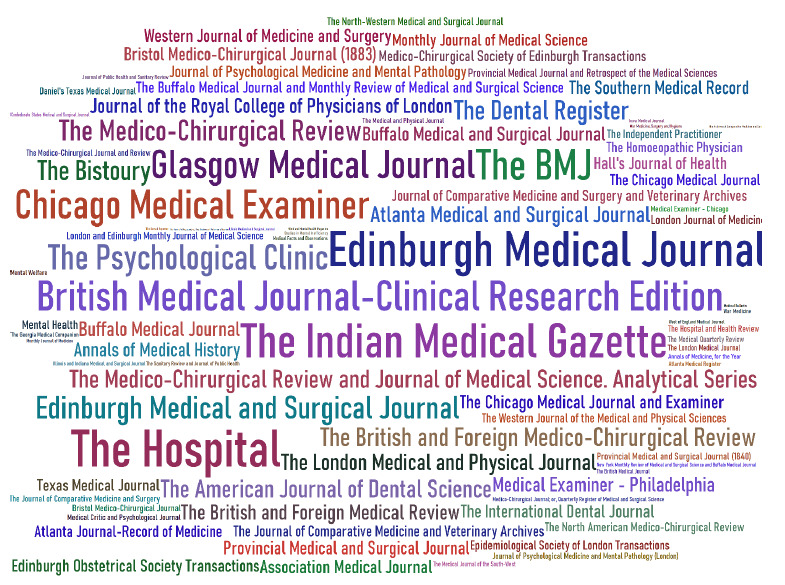
Depicting all titles of PubMed Central (PMC) Journal Backfiles Digitization available under a Creative Commons License or in the Public Domain, and located at https://ftp.ncbi.nlm.nih.gov/pub/pmc/historical_ocr/. Title sizes are proportional to individual file sizes which range from 0.2 to 116 megabytes.

**Figure 2:**
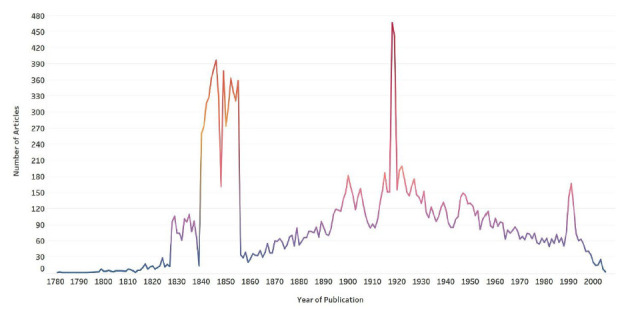
Depicting all titles of Figure 1 by number of articles and year of publication.

From 2004 to 2010, the project included journals both already participating in and just getting started with PMC, whose back issues were not yet available in digital form. It involved a multidisciplinary team comprised of staff of the National Library of Medicine (NLM), Wellcome Trust, and UK Joint Information Systems Committee (JISC). Together, they embarked on a process of sourcing the complete physical runs of participating journals, digitizing them, and linking their associated data. Among the journals included in the project were the *Annals of Surgery*, *Biochemical Journal*, *Journal of Physiology* and *Medical History*, and many more which illuminate the intersection of science and society over two centuries [3].

The process involved more than simply scanning pages. The NLM team of technical experts meticulously tagged each article with metadata, including titles, authors, affiliations, and publication details. The team created a PDF file for every discrete item in the archive (article, editorial, letter, advertisement, etc.), and they used optical character recognition (OCR) technology to generate searchable text. They developed innovative ways to link articles with corresponding records in the catalog of the NLM, as well as Digital Object Identifiers (DOIs) when available, making more content discoverable and readily available to researchers, teachers, and learners worldwide. Additionally, the team created corresponding PubMed records from the scanned articles that had not already been indexed in PubMed to further increase discoverability and availability of this content.

By the end of 2010, the team made available, through PMC, more than one million articles from 160 journals, spanning nearly two centuries. This corpus included groundbreaking works that changed the face of medicine, such as Alexander Fleming's 1929 paper on penicillin, Richard Doll's 1954 study linking smoking to lung cancer, and Walter Reed's 1902 research demonstrating mosquito transmission of yellow fever [2, 3].

The impact of this newfound online availability was immediate and profound. The *Journal of Anatomy*, recognizing the value of open access, celebrated the availability of its entire archive online, noting its inclusion in the “100 most influential journals in biology and medicine over the past 100 years [4].” Similarly, *Medical History* lauded the project's alignment with open access principles, making its entire archive freely available for the first time [5]. Comparable appreciation of the PMC backfiles initiative appeared in the *Biochemical Journal*, *British Journal of Medical Practice*, *Canadian Medical Association Journal*, and *Journal of Physiology* [6, 7, 8, 9]

Building on this success, and in continued partnership with the Wellcome Trust, PMC Journal Backfiles Digitization expanded its scope from 2014 to 2019 [10]. The focus shifted to include the physical preservation of the original journals in their printed and published form, ensuring their long-term survival for future generations. Work began with journals related to mental health, including *Mental Health*, *Mental Hygiene*, *Journal of Psychological Medicine* and *Mental Pathology*. The project broadened its selection to include journals of general relevance, such as the *British and Foreign Medico-Chirurgical Review* and *Transactions of the Epidemiology Society of London*. It also encompassed titles long sought after in digital form by historians, such as the *Indian Medical Gazette* and *The Hospital* [11, 12].

By 2019, the team added over two dozen titles to PMC, spanning three centuries, encompassing hundreds of thousands of pages, and including certain titles determined to be Orphan Works. The result was a vast searchable collection of over 650,000 pages comprising tens of thousands of articles, all freely available for reading, downloading, and even text mining. Articles from Orphan Works appeared under the Creative Commons Attribution-NonCommercial 4.0 International License. Today and tomorrow, countless new avenues for discovery and understanding await all researchers, teachers, and learners who explore PMC and study its rich historical content.

Indeed, engagement with this corpus was remarkable. On average, 150,000 articles from the collection were viewed each month. In 2024 alone, users downloaded the historical optical character recognition (OCR) dataset, consisting of nearly 90 titles, in its entirety approximately 300 times. Historical articles made available under Creative Commons license terms are also integrated into the PMC Open Access Subset, a rich corpus of more than six million biomedical research articles available for bulk retrieval and reuse [13]. While the specific uses of the downloaded data remain unknown, the sheer volume of downloads suggests a high level of interest and utilization by researchers, teachers, and learners.

Recognizing the importance of continuous outreach to and engagement with NLM's patrons, the project team combined their experience and expertise in various strategies to highlight the value of the collection for research, teaching, and learning. They co-authored blog posts on popular platforms including *Circulating Now*, *History News Network*, and *Musings from the Mezzanine*, thereby showcasing the content of specific journals and encouraging their exploration and discovery. They also worked together to create thematic visualizations of the corpus, by creating compelling snapshots of its wealth of information ripe to be studied [14, 15, 16].

The next chapter of PMC Journal Backfiles Digitization, spanning from 2019 to early 2024, focused on digitizing public domain titles, notably the *Atlanta Journal-Record of Medicine* (1899-1918), *Buffalo Medical Journal* (1895-1918), *The Chicago Medical Journal and Examiner* (1875-1889), *The Dental Register* (1847-1923), and *Daniel's Texas Medical Journal* (1893-1919). This effort added to PMC nearly two dozen more titles and over 75,000 articles, further expanding its depth and breadth [17].

PMC Journal Backfiles Digitization stands as a testament to the transformative power of open access and digital preservation. By making a vast collection of historical medical literature freely available, the project has democratized access to this knowledge, opening to researchers, teachers, and learners countless new avenues for understanding medicine as it has changed over time, and its changing impact on science and society.

PMC Journal Backfiles Digitization also highlights areas for future growth, development, and engagement with researchers, teachers, and learners in the related and evolving fields of digital humanities, data science, and artificial intelligence. Indeed, through further advancements in AI and text-mining techniques, opportunities abound to explore and learn from this open access corpus. Term proximity searches hold the promise of identifying early mentions or implicit references to medical conditions before their larger recognition, while sentiment analyses could identify changes in opinion related to treatments, public health policies, and emerging medical technologies.

The team acknowledges the need to understand better how researchers, teachers, and learners have utilized the corpus, and how many more could benefit from its content. Direct user surveys could provide valuable insights into the impact of this open access. They could also inform future digitization efforts and priorities. The co-authors of the article certainly welcome hearing feedback from individuals who are studying PMC backfiles to unearth new insights about the human condition across time and place.

The success of PMC Journal Backfiles Digitization provides a blueprint for future efforts to make NLM's globally appreciated historical collections accessible to all. By continuing to prioritize open access and leverage lessons learned in digitization initiatives, NLM can ensure that knowledge and data across historical time and place continue to inform the advancement of medicine and public health across time and place today and in the future.
